# A Template-Based Protein Structure Reconstruction Method Using Deep Autoencoder Learning

**DOI:** 10.4172/jpb.1000419

**Published:** 2016-12-12

**Authors:** Haiou Li, Qiang Lyu, Jianlin Cheng

**Affiliations:** 1 Department of Computer Science and Technology, Soochow University, Suzhou, 215006, China; 2 Department of Computer Science, University of Missouri, Columbia, MO 65211, USA

**Keywords:** Protein structure prediction, Template-based modeling, Deep learning, Deep autoencoder

## Abstract

Protein structure prediction is an important problem in computational biology, and is widely applied to various biomedical problems such as protein function study, protein design, and drug design. In this work, we developed a novel deep learning approach based on a deeply stacked denoising autoencoder for protein structure reconstruction. We applied our approach to a template-based protein structure prediction using only the 3D structural coordinates of homologous template proteins as input. The templates were identified for a target protein by a PSI-BLAST search. 3DRobot (a program that automatically generates diverse and well-packed protein structure decoys) was used to generate initial decoy models for the target from the templates. A stacked denoising autoencoder was trained on the decoys to obtain a deep learning model for the target protein. The trained deep model was then used to reconstruct the final structural model for the target sequence. With target proteins that have highly similar template proteins as benchmarks, the GDT-TS score of the predicted structures is greater than 0.7, suggesting that the deep autoencoder is a promising method for protein structure reconstruction.

## Introduction

Protein Data Bank (PDB) is a database that provides rich structural information of many proteins. The structures of these proteins were mainly determined by X-RAY or NMR technology. Up to January 2016, the database had 106,554 known protein structures. However, compared with the known primary sequence, the number of known protein structures is still much fewer than the number of known sequences [1]. This suggests that using experimental methods can only determine the structures of a tiny portion of all the protein sequences due to their relatively high cost. So computationally predicting protein structures is very important to address this sequence-structure gap.

One useful component of protein structure prediction is protein structure reconstruction (or generation) [2,3]. Given the preliminary or coarse-grained structural information of a target protein (e.g., the structure of a homologous protein whose sequence is similar to the target protein), the reconstruction is to generate a more accurate structural model for the target protein. PULCHRA [4] is a method that uses Cα atoms and the center of side-chain mass coordinates to reconstruct full-atom protein models. Peng et al. developed a method that determines the positions of some key heavy atoms (N, C, O and side chain C_β_) based the knowledge of the Cα atom coordinates [5]. Both of these two protein structure reconstruction methods depend on the accuracy of Cα atoms. Baeten et al. also introduced a method that reconstructs protein backbones with protein fragments [6].

Here, we developed a new deep learning method for protein structure reconstruction. Recently, deep learning has been used in protein secondary structure prediction [7,8], protein fold prediction [9–11] and protein contacts prediction [12,13]. In this study, we combined deep learning with the templates searched from big protein structure database to reconstruct protein structures for template-based protein structure modeling.

Template-based modeling is one of the main approaches to protein structure prediction. The principle of template-based protein structure is based on the following insights [14]: Proteins that have similar sequences (e.g., homologous proteins) often have similar tertiary structures. Therefore, we can use the structure of a protein’s homolog as the template to predict its structure. It is also shown that the conservation of the tertiary structure is much greater than that of the primary sequence [15]. Therefore, if similarity between two proteins is significant at the sequence level, their structural similarity can usually be assumed.

Most current template-base modeling methods consist of four steps: (1) Search homologous templates for a target sequence; (2) Align the template sequences with the target sequence to generate an alignment; (3) Build models for the target sequence according to the alignment and the structures of the template proteins; and (4) Evaluate and rank the models [1,11,16,17]. During the last few decades, a lot of progress has been made in template-based modeling [2,18,19]. SWISS-MODEL [20] generates a core model by averaging template backbone atom positions. NEST [21] implements an artificial evolution algorithm where changes from the template structure such as substitutions, insertions and deletions are made at one time, and each mutation is followed by energy minimization. This process is repeated until the whole target protein is modelled. MODELLER [22] is one of the most popular template-based protein modeling programs that use spatial constraints. In MODELLER, the various spatial relationships of distances and angles are expressed as conditional probability density functions and are then used directly as spatial restraints to build models. I-TASSER [23] constructs 3D structural models by reassembling fragments excised from threading templates starting from the amino acid sequence. The accuracy of all of these methods depends on the quality of templates and alignments.

In this work, we focus on using structural data of templates to infer the 3D structure of the target sequence. We present a protein structure reconstruction method that is based on a deep stacked denosing autoencoder (named PRSDA). The method only relies on the tertiary coordinates of the templates of a target protein. It trains a deep autoencoder model by using a large set of template model decoys as a training set to reconstruct the structure of the target protein.

## Results and Discussion

In order to assess the reconstruction accuracy of our method we performed two tests on two different testing data sets. We compared our method with MODELLER and I-TASSER–two classic template-based protein structure prediction methods. In the first test, we want to validate whether or not PRSDA could reconstruct protein structures successfully. The second test shows what kind of target sequences suitable for the PRSDA method. We tried to use unsupervised pre-training to initialize the parameters of the deep learning model. Compared with the experiments of random initialization without pre-training, it slightly improved the accuracy on some targets tested. For instance, we tried different amounts of pre-training data points (i.e., 200, 400 and 800) and observed CRMSD difference less than 0.1 on some targets tested. We used layer-wised pre-training on the whole training dataset to initialize the parameters of the network for them.

We chose 14 testing targets and obtained useful information on each one, such as target sequence, best template, GDT score, and CRMSD score in Gront et al. [24]. In order to objectively compare our PRSDA method and MODELLER, we applied the methods to the same best template for each of the targets and compared the final modeling results. In this way, we used our PRSDA method as single-template protein structure reconstruction method.

The comparison result between MODELLER and PRSDA in terms of GDT score and CRMSD (i.e., RMSD of Ca atoms) is shown in Table 1. Columns under ‘PRSDA’ are best results reconstructed by PRSDA. And columns under ‘MODELLER’ are best results of MODELLER in Gront et al. [24]. GDT score is considered more robust against local structural difference than RMSD, and is a major assessment metric used in the Critical Assessment of Techniques for Protein Structure Prediction (CASP) [25]. In Table 1, PRSDA performed worse than MODELLER on seven targets in terms of CRMSD. But except for target “3DTO”, their GDT scores have no significant differences. We made a paired t-test on the GDT scores of the 14 predictions made by PRSDA and MODELLER, assuming H_0_: µ_d_ =0, H_1_: µ_d_ ≠ 0, α =0.05, i.e., there is no difference between the GDT scores. The P-value of two-tail t-test is 0.10 (>α), suggesting that the performance of PRSDA is comparable to MODELLER on these targets. For the target “3DTO”, its native structure has a lot of flexible loops, which is hard for our method to predict. The results in Table 1 demonstrate that our deep learning architecture can use the spatial coordinates of templates to reconstruct the structures of target proteins effectively on this dataset.

In the training data set we generated templates’ structures using 3DRobot, some of those structures were close to the templates, but some deviated significantly. Because the input structures vary a lot, the modeling can be trained to get a common rule for the testing data sets. So the input testing data set can be reconstructed to a new output. If the template is not far away from the target structure, then the output should be close to the true target structure.

The second test focused on comparing PRSDA with MODELLER and I-TASSER (two of the top template-based modeling methods) on target proteins whose sequences have high sequence similarity with template proteins. In order to carry out the test, we installed MODELLER and I-TASSER on our local server. We ran them with default parameter values separately to obtain top 5 models for each target.

The results of the comparison are shown in Table 2. PRSDA predicted highly accurate structures with RMSD less than 1 Angstrom for 15 out of 21 targets, MODELLER 14 out of 21 targets, and I-TASSER 8 out of 21 targets. MODELLER produced the best models for more than half of the targets. We conducted a paired T-test to check whether there is significant difference between MODELLER and other two methods. Our hypothesis is there is no significant difference between MODELLER and the other method (H_0_: µ_d_ =0, H_1_:µ_d_ ≠ 0, α =0.05). The p-value of two-tail test between the MODELLER and PRSDA is 0.089, which is greater than α, suggesting that the performance of these two methods has no significant difference. But p-value of the t-test between I-TASSER and MODELLER is 0.000173, suggesting their difference is significant.

**Figure 1:**
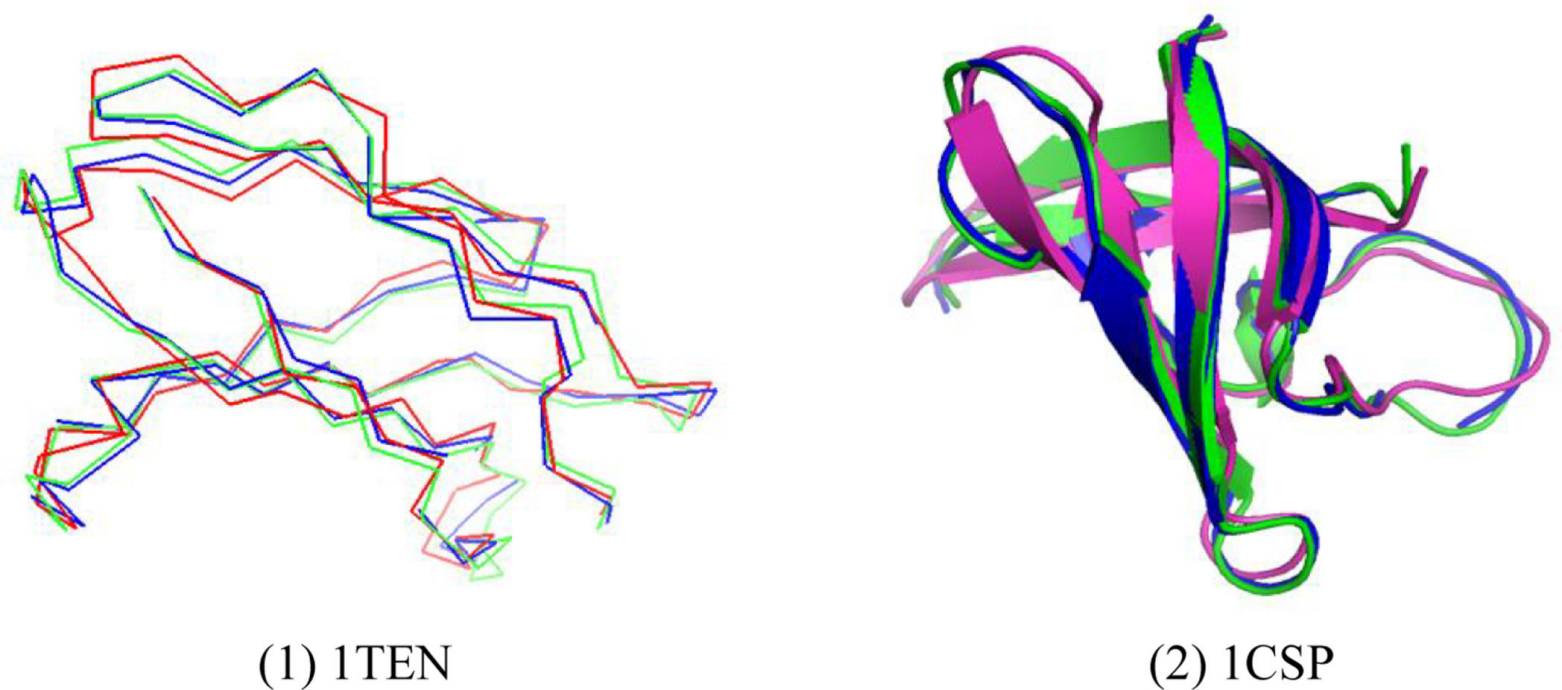
Comparision of predicted structures and their native structure in chain conformation (1) and cartoon conformation (2). For the two typical pdbs, native structure, reconstructed structure and predicted structure from I-TASSER are represented in green, blue and red color respectively.

All of the three methods performed very well on most of the target sequences. Figure 1 illustrates one case in which PRSDA performed better than I-TASSER. The left part of Figure 1 compares the structure predicted by PRSDA, I-TASSER and the native structure of target 1TEN. We used PyMOL [26] to visualize the structures. On this target, the structure predicted by PRSDA (the blue line) can cover more areas of the native structure (the green line) than the structure predicted by I-TASSER (the red line). The right part of Figure 1 compares the structures of PRSDA (the blue one), I-TASSER (the red one) and the native structure (the green one) in the cartoon mode.

**Figure 2:**
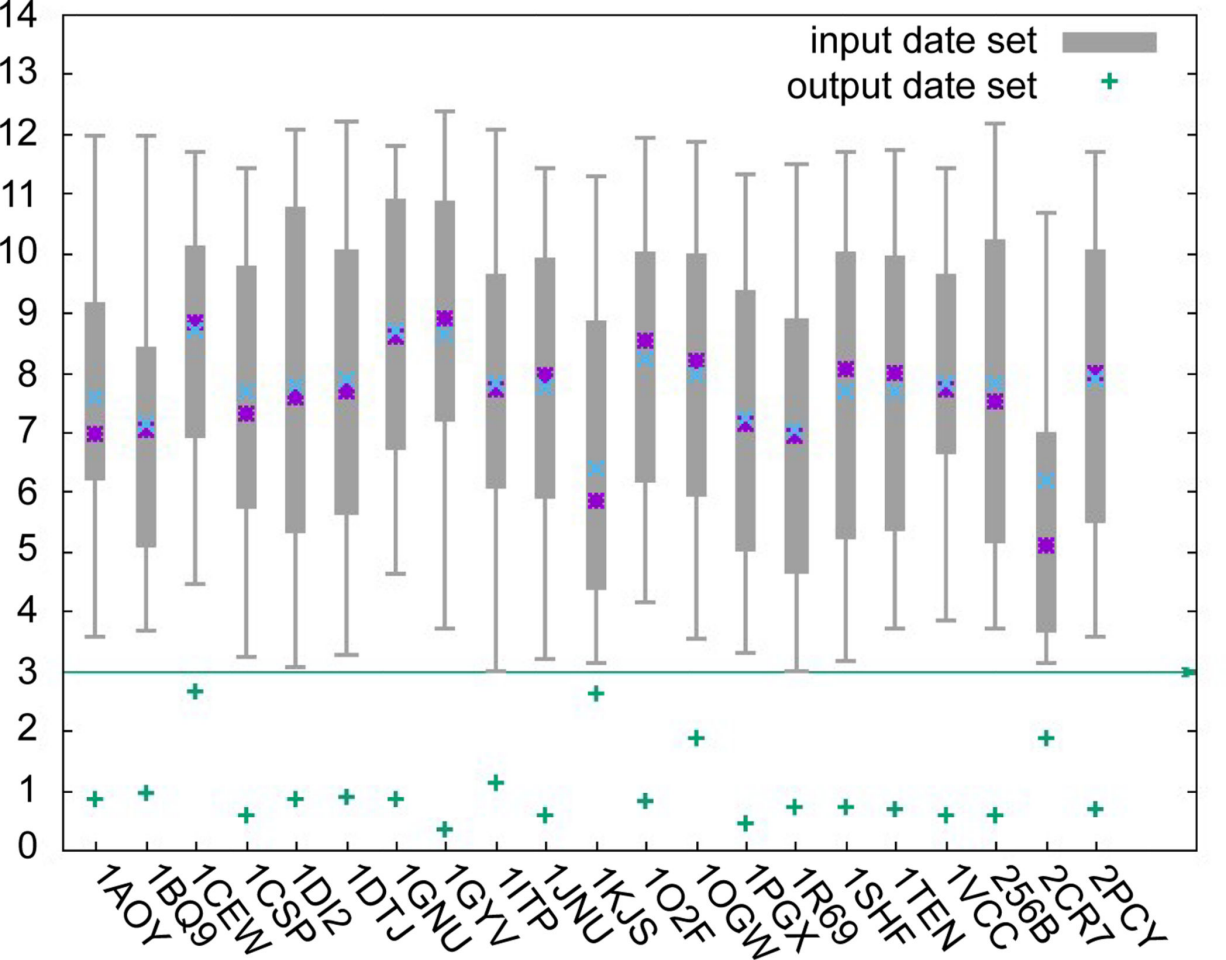
The data statistic of input data sets and output data set. The box-and-whisker diagram shows the RMSD distribution between input conformation and native conformation, the blue and purple points represents median and average values. Points plotted in green color represent the average RMSD between reconstruction conformation and native conformation.

In order to further validate the robustness of our method, we prepared extra testing data sets for each target. The RMSD (comparison of estimation error) of input testing datasets varied from 3 to 13 Å (as shown by the data distribution in Figure 2). But the RMSD of all generated output structures was lower than 2 Å (the green cross points). On 15 of the whole 21 targets, the RMSD of their output structures was lower than 1 Å. From the output data sets, we found that the RMSD of output structures is almost same on each target, which means the variation of input testing dataset, do not significantly affect the output in general.

**Figure 3:**
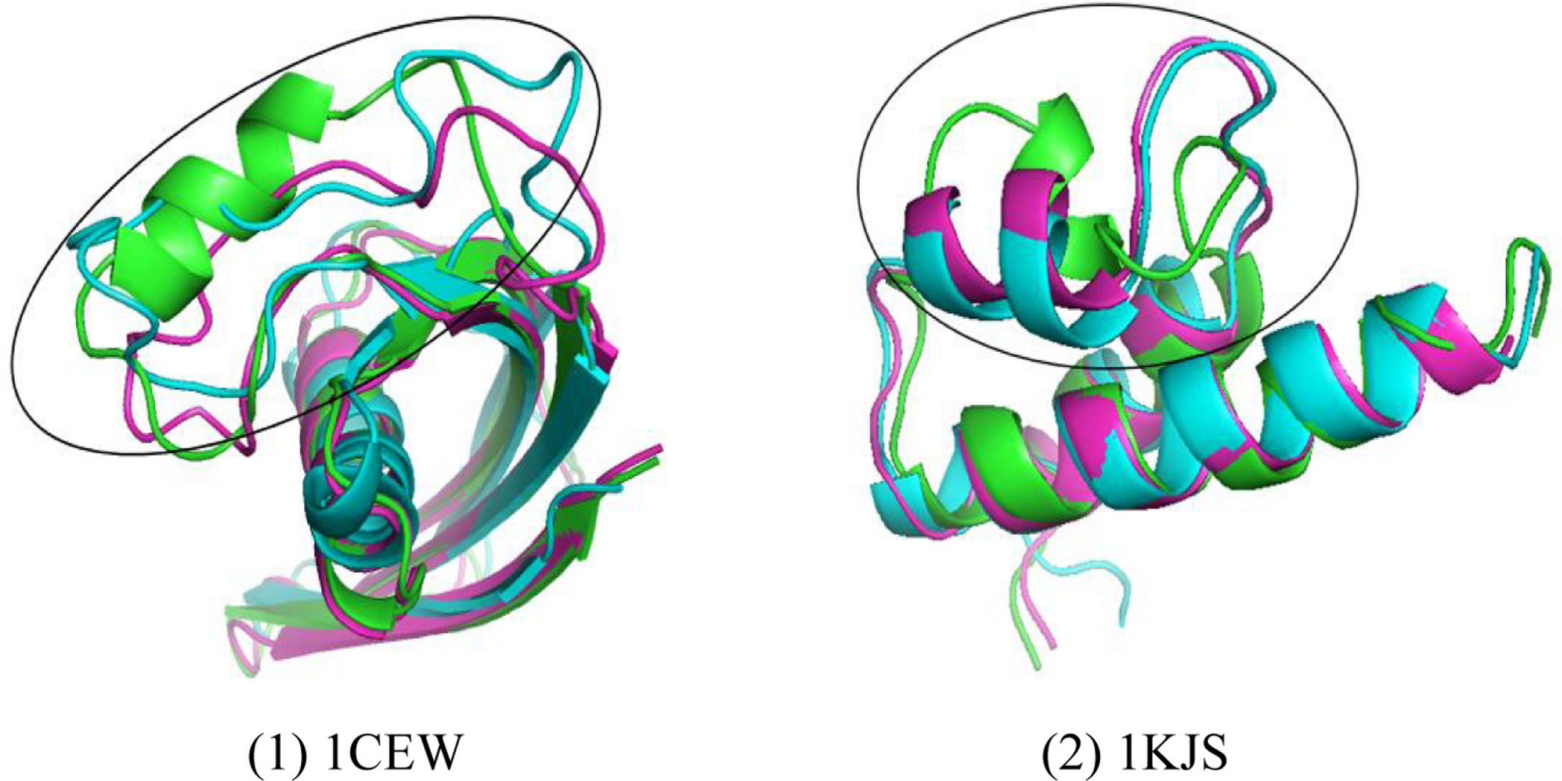
Templates for target (1) 1CEW and (2) 1KJS. For the two typical pdbs, native structure, and templates structure for the targets are represented in green and other colors (blue and rose red) respectively.

However, PRSDA failed to predict accurate models for two targets (1CEW and 1KJS). This was due to the structural discrepancy between the native structure of the target protein and the structure of the templates. Figure 3 shows the templates of target 1CEW and 1KJS and their native structure separately. The green one represents the native structure, the blue and red ones represent the two templates. A big difference is observed between the structure of the target protein and those of the template proteins.

In summary, our PRSDA method is a new deep learning method applied to protein structure reconstruction. The PRSDA method is different from some traditional protein structure prediction methods, in that it does not need some intermediate structural information such as secondary structure, solvent accessibility, or fragments. Our deep autoencoder learning method can learn some useful information from template structures. The current method performs comparably with two state-of-the-art protein structure modeling methods when the sequence similarity between target proteins and templates is high.

## Methods

### Data sets and evaluation metrics

We used two testing data sets to evaluate our method. The first testing data set was curated from the data set in Gront et al. [24], used to assess the accuracy of a template-based structure prediction method-MODELLER. It contains 14 protein sequences whose length varied from 90 to 509 from the data set [24]. The second testing data set is from (a set of targets used by I-TASSER), filtering out some sequences that do not have any sequence similarity with templates according to the PSI-BLAST search [27,28]. We finally obtained 21 testing targets, all of which had a sequence similarity with their templates proteins ≥50%. The details about how to prepare the training and testing data sets for our deep learning are explained in the section entitled “Training of Autoencoder for Protein Structure Reconstruction”.

The Root-Mean-Square Deviation (RMSD) is a measure of the average distance between the atoms of superimposed protein structures. For each reconstructed decoy we calculated its backbone RMSD and Cα RMSD with respect to the native structure of its corresponding target protein. The lower the RMDS value is, the better the decoy model is. Since RMSD is a length-dependent measure method, we also used another evaluation metric referred to as the Global Distance Test (GDT) to assess the quality of decoys. GDT measures the fraction of a structural model that can be superimposed to its native structure within a given distance. The GDT value varies from 0.0 to 1.0 and the higher the better.

### Deeply stacked denoising autoencoder

The deep autoencoder is a popular unsupervised machine learning method, which can make nonlinear dimension reduction on original data in order to obtain the low-dimension data to represent the high-dimensional data. The Denoising Autoencoder (DA) is a kind of special autoencoder, which adds some noise on the input data to improve robustness [29]. A Stacked Denoising Autoencoder (SDA) stacks multiple layers of DA together to improve the accuracy of dimension reduction [30].

**Figure 4:**
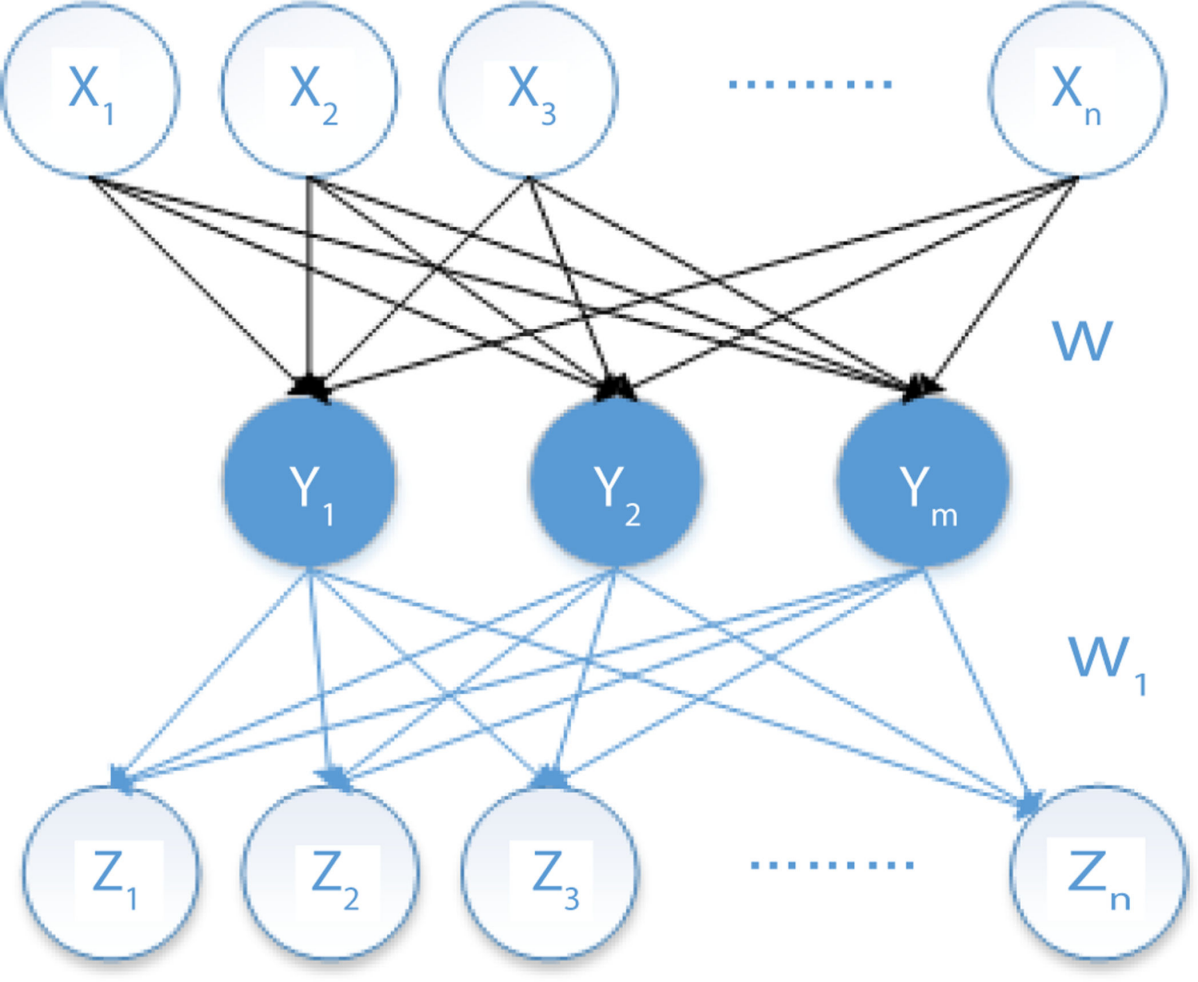
The basic architecture of an autoencoder.

Figure 4 denotes a basic model of an autoencoder. The first layer (X_1_, X_2_, …, X_n_) represents the input, the middle layer (Y_1_, Y_2_, …, Y_m_) is the hidden layer, and the last layer (Z_1_, Z_2_, …, Z_n_) is the output. The number of nodes on each layer can be adjusted according to the complexity of the problem.

The mapping formula from the input layer *x* ∈ (0,1) to the hidden layer *y* ∈ (0,1) is:

(1) $$y=S(W_{x}+b)$$

Here, *S* is the sigmoid function as follows:

(2) $$\text{Sigmoid}(x)=\frac{1}{1+e^{-x}}$$

The mapping between hidden layer y ∈ (0,1) and the output layer z ∈ (0,1) is calculated by a similar function as follows.

(3) Z=W′y+b′

In these two mapping relations, W, b, b’ are the weights of the autoencoder. W is a matrix of N*M, where *N* is the dimension of the input data and M is the dimension of the hidden layer data. b is a vector with length of M, b’ is a vector with length N, and W*’* is the transposed of matrix W. z is reconstructed data of x. The objective is to make z as consistent with x as possible.

### Stacked denoising autoencoder for protein structure reconstruction

**Figure 5:**
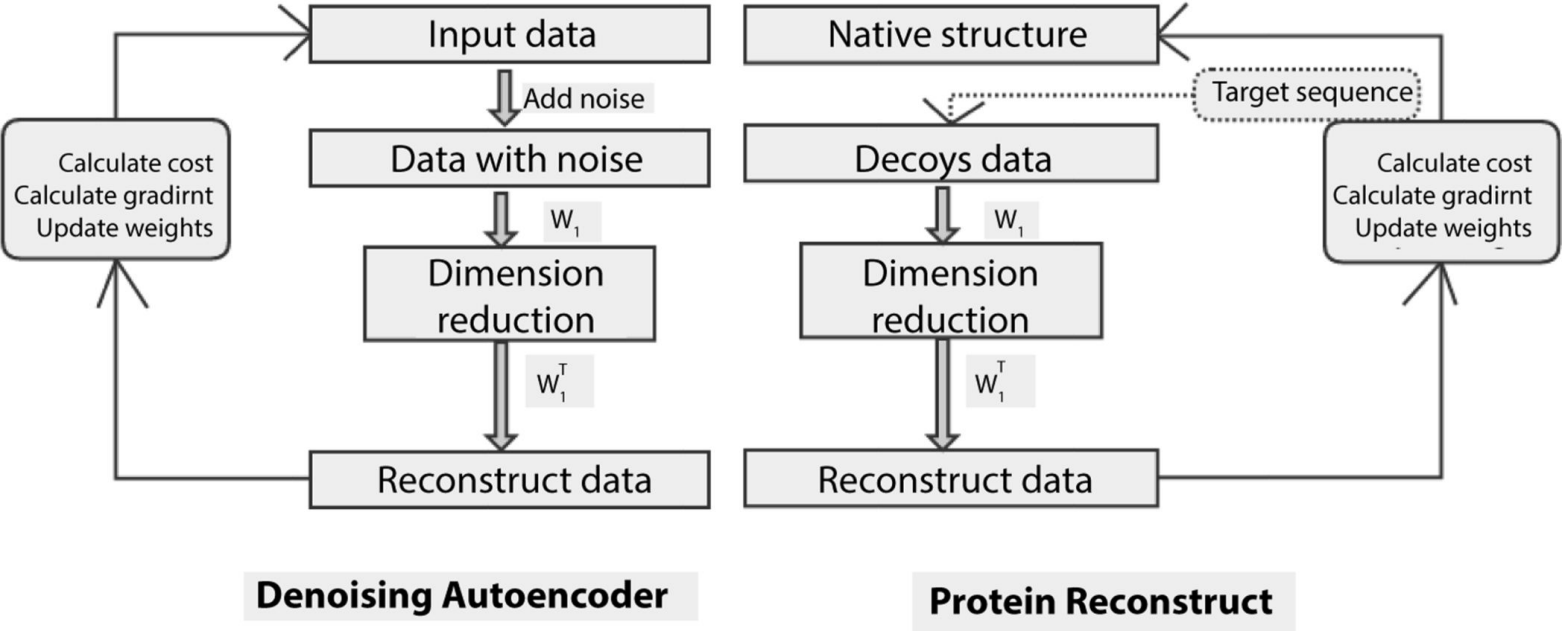
The architecture of denoising autoencoder for protein structure reconstruction. The left part is the principle of denosing autoencoder, and the right part is the principle of protein structure reconstruction.

Inspired by the successful example of using autoencoder for handwritten digital number recognition, we designed a deep learning model based on the denoising autoencoder to reconstruct protein structure (Figure 5). In the training phase, the templates’ native structures are considered pure data without noise. The predicted protein decoys can be thought of as noisy data. During training of the denoising autoencoder, the native structures of those templates are known in advance to tune its parameters. In the prediction phase, the trained model can be applied to the untrained target proteins whose native structures are not yet known, but are close enough to those of the training proteins.

### Representation of structure in a deep autoencoder model

In this work, we used x, y, z coordinates of backbone atoms to express protein spatial structure. When using RMSD, it is easy to measure the gap between reconstruction data and label data. We used the coordinates of backbone atoms and residue symbol as the input data for the deep learning model. Before the data coordinates of input structures are converted into input matrix, they are superimposed and translated to be in the same coordinate system.

Because an initial coordinate is often outside the range of [0,1], we use the following linear function to normalize it into the range of (0, 1).

(4) $$x_{\text{new}}=\frac{x-x_{\text{min}}}{x_{\text{max}}-x_{\text{min}}}(x\varepsilon X)$$

*X* represents a set of coordinates. *x* is an initial coordinate. *Xmax* and *Xmin* are the maximum and minimum coordinates in *X*. Assuming these are the initial coordinates of two atoms: (*x_1_, y_1_, z_1_*), (*x_2_, y_2_, z_2_*), the original distance between them is as follows:

(5) $$L=\sqrt{{\left(x_{1}-x_{2}\right)}^{2}+{\left(y_{1}-y_{2}\right)}^{2}+{\left(z_{1}-z_{2}\right)}^{2}}$$

The compression ratio after normalization is *N*,

(6) $$N=X_{\text{max}}-X_{\text{min}}$$

After compression, the two atoms coordinates are:

(7) $$\left(\frac{x_{1}}{N},\frac{y_{1}}{N},\frac{z_{1}}{N})\;\text{and}\;(\frac{x_{2}}{N},\frac{y_{2}}{N},\frac{z_{2}}{N}\right)$$

Then the distance of two atoms after compression are as follows:

(8) $$L=\sqrt{{\left(\frac{x_{1}}{N}-\frac{x_{2}}{N}\right)}^{2}+{\left(\frac{y_{1}}{N}-\frac{y_{2}}{N}\right)}^{2}+{\left(\frac{z_{1}}{N}-\frac{z_{2}}{N}\right)}^{2}}\;=\frac{1}{N}\sqrt{{\left(x_{1}-x_{2}\right)}^{2}+{\left(y_{1}-y_{2}\right)}^{2}+{\left(z_{1}-z_{2}\right)}^{2}}$$

This means the relative distance between two atoms shrinks by the same ratio as normalization. Therefore, the normalized new dataset can represent the original dataset well.

### Training of autoencoder for protein structure reconstruction

We used RMSD to measure the difference between the coordinates of a native structure and those of a predicted structural model as objective function to guide the training of the autoencoder as follows:

(9) $$\text{RMSD}=\sqrt{\frac{\sum_{k=1}^{n}{\left(X_{k}-x_{k}\right)}^{2}+{\left(Y_{K}-y_{k}\right)}^{2}+{\left(Z_{k}-z_{k}\right)}^{2}}{n}}$$

Where, X_k_, Y_k_, Z_k_ represent native structure coordinates; x_k_, y_k_, z_k_ are the predicted structural coordinates, and n is the number of total atoms. We implemented the calculation of RMSD in Theano using GPU-Q-J, which use GPU to accelerate the calculation [31].

**Figure 6:**
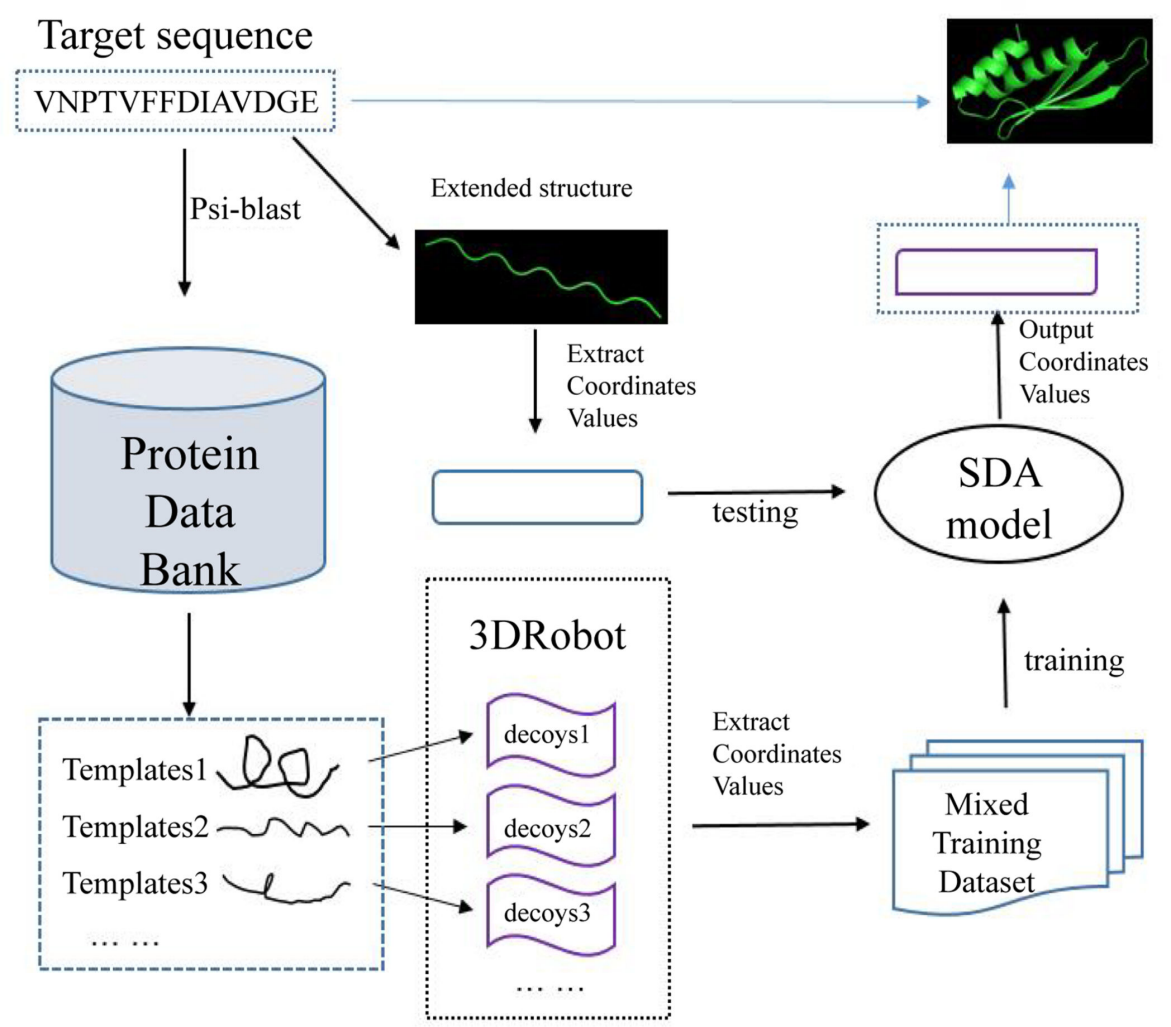
Flowchart of the protein structure reconstruction method.

We designed a deep learning model based on the stacked denoising autoencoder for protein structure reconstruction (PRSDA). In order to predict the tertiary structure of a protein target sequence, we used its homology structures to train the weights of the PRSDA model, and then test the model on a preliminary structure from the target sequence; finally we can get the reconstructed structure for the target sequence. Figure 6 shows the flowchart of PRSDA methodology.

According to Figure 6, the input to our method is a target sequence, and the output is a reconstructed protein structure. Specifically, our method has the following six steps.

1. Prepare appropriate templates for the target sequence: This step is to use PSI-BLAST to find templates for the target sequence, and then filters out those templates that have low sequence similarity with the target sequence. We align the templates sequences to the target sequence and make them have a consistent length with the target sequence. We get rid of extra parts of a template if it is longer than the target sequence. The vacant parts are filled with 0.
2. Generate decoys for the templates: The second step is to use 3DRobot [32] to generate decoys for the templates. Generally, we generate 500 decoys for each template, but if there is only one appropriate template for the target sequence, we generate 1000 decoys for this template. The input of 3DRobot is a protein PDB file and the outputs are diverse and well-packed protein structure decoys. 3DRobot generates a lot of diverse models with a wide range of RMSD compared to the input PDB structure. Some of these generated decoys are close to the input, but some of them are far away from the input.
3. Representation of training dataset: After template decoys are generated, we extracted the coordinates of their 4 backbone atoms (C, Cα, N, and O) to prepare training datasets. Each atom has 3 coordinates (X-axis, Y-axis, and Z-axis). So for each residue, there are 12 numbers to represent its spatial position. The total number of the input features for the deep learning model is L*(12+1), where L is the length of the target sequence. The extra input represents the amino acids type. The labels of training datasets are the coordinates of templates’ native structures.
4. Construct the architecture of the training model: We used these training datasets to train the deeply stacked autoencoder model. The first layer is a visible layer and the node number of this layer equals to the number of the input features (L*(12+1)). Next to the visible layer, there are three hidden layers. The number of the hidden layers’ nodes is reduced layer by layer, and the decrease ratios are 0.9, 0.8, and 0.7 respectively. After training, we save the parameters of the model for predicting structure of the target sequence.
5. Testing the model on a target sequence: First, we set torsion angles (phi, psi and omega) for each residue of the target sequence to +135°, −135° and 180°, and then we use Rosetta [33] to convert torsion angles to X, Y, Z coordinates to get an extended structure. So the input data of the testing process is the coordinates from the extended structure and the output of the model are new coordinates values for the target sequence.
6. Construct PDB file for the target sequence: Finally, we combine these new coordinate values and target sequence information to construct a standard PDB file as the final protein reconstruction structure.

## Figures and Tables

**Table 1:** The results comparison of PRSDA and MODELLER on the first dataset.

Experimental information				PRSDA		MODELLER	
Target	Length	Template^a^	Similarity^b^	GDT(2)^c^	Crmsd^d^	GDT(2)^c^	Crmsd^d^
3D3N	247	3BXP	92%	0.8947	2.37	0.906	2.68
3DBI	269	3BRQ	86%	0.7361	2.778	0.704	2.71
3D4O	278	2RIR	63%	0.5683	2.897	0.555	2.77
3DO6	509	1FPM	54%	0.9136	1.676	0.919	1.31
3CKW	231	3COM	51%	0.9091	1.567	0.863	1.48
3DTO	180	2PJQ	46%	0.4889	5.571	0.810	3.03
3D7Q	101	2NLV	45%	0.9208	1.523	0.929	1.35
3DJB	174	2QGS	44%	0.7931	3.12	0.819	2.18
3D3U	360	2OAS	43%	0.8667	2.048	0.918	1.72
3DA1	462	2RGO	43%	0.7684	4.037	0.849	2.01
3DFH	352	3BSM	40%	0.8920	1.567	0.962	1.3
3CTV	90	3HDH	39%	0.6111	9.217	0.772	3.04
3DO5	277	2EJW	37%	0.6679	5.124	0.653	2.82
3DMB	142	2QEA	34%	0.5704	5.011	0.628	3.07

a The best template for the target sequence

b The sequence similarity between target sequence and best template

c The GDT Score of the reconstruct structure compared with the native structure

d The Cα RMSD of the best predicted structure compared with the native structure

**Table 2:** The results of PRSDA, MODELLER and I-TASSER on the second dataset.

Experiment information		PRSDA		MODELLER		I-TASSER	
Target	Length	GDT-TS^a^	Crmsd^b^	GDT-TS^a^	Crmsd^b^	GDT-TS^a^	Crmsd^b^
1AOY	62	0.9597	0.765	0.9919	0.585	0.9274	1.195
1BQ9	52	0.9952	0.501	0.9952	0.469	0.9904	0.613
1CEW	108	0.7361	2.606	0.8704	2.74	0.7986	2.447
1CSP	65	0.9697	0.633	0.9962	0.475	0.9053	1.19
1DI2	69	0.9638	0.802	0.9746	0.676	0.9783	0.614
1DTJ	73	0.9692	0.924	0.9658	1.533	0.9623	0.818
1GNU	115	0.9717	0.844	0.9804	0.799	0.8913	1.261
1GYV	119	0.9979	0.330	0.9958	0.456	0.9328	0.621
1ITP	68	0.9154	1.128	0.9228	1.079	0.9384	0.987
1JNU	97	0.9871	0.637	0.9897	0.583	0.9201	1.203
1KJS	55	0.7045	2.724	0.7545	2.83	0.7455	3.198
1O2F	68	0.9816	0.703	0.9853	0.66	0.9338	0.873
1OGW	74	0.9595	1.824	0.9392	1.923	0.9054	1.837
1PGX	56	0.9955	0.520	0.9955	0.501	0.942	0.938
1R69	69	0.9637	0.767	0.9597	0.97	0.9274	1.111
1SHF	59	0.9958	0.503	0.9958	0.491	0.9534	1.186
1TEN	89	0.9775	0.745	0.5337	3.761	0.5309	3.677
1VCC	77	0.9903	0.589	0.9968	0.527	0.9513	1.045
256B	106	0.9858	0.568	0.9976	0.46	0.9316	1.26
2CR7	60	0.7421	2.129	0.877	1.465	0.9127	1.275
2PCY	99	0.9823	0.656	0.9924	0.485	0.852	0.905

a The GDT-TS score of the best predicted structure compared with the native structure

b The Cα RMSD of the best predicted structure compared with the native structure
